# Oral Health and Hygiene and Association of Functional Ability: A Cross-Sectional Study Among Old Home Care Clients

**DOI:** 10.3290/j.ohpd.a43353

**Published:** 2020-07-04

**Authors:** Eveliina Tuuliainen, Kirsi Autonen-Honkonen, Annamari Nihtilä, Kaija Komulainen, Irma Nykänen, Sirpa Hartikainen, Riitta Ahonen, Miia Tiihonen, Anna-Liisa Suominen

**Affiliations:** a PhD Student, Institute of Dentistry, University of Eastern Finland, Yliopistonranta 1, FI-70210 Kuopio, Finland. Wrote the manuscript, performed statistical analyses.; b Dental Hygienist, Public Health Center, Oral Health Care Services, City of Jyväskylä, Finland. Proofread and critically revised the manuscript, experimental design.; c University Lecturer, Institute of Dentistry, Department of Oral and Maxillofacial Surgery, Kuopio University Hospital, Yliopistonranta 1, FI-70210 Kuopio, Finland. Cowrote and critically revised the manuscript.; d PhD Student, Institute of Dentistry, University of Eastern Finland, Yliopistonranta 1, FI-70210 Kuopio, Finland. Cowrote and critically revised the manuscript.; e Professor, Kuopio Research Centre of Geriatric Care, Institute of Public Health and Clinical Nutrition, School of Pharmacy, Faculty of Health Sciences, University of Eastern Finland, Yliopistonranta 1, FI-70210 Kuopio, Finland. Proofread and critically revised the manuscript, experimental design.; f Professor, Kuopio Research Centre of Geriatric Care, School of Pharmacy, Faculty of Health Sciences, University of Eastern Finland, Yliopistonranta 1, FI-70210 Kuopio, Finland. Proofread and critically revised the manuscript. Experimental design.; g Professor, School of Pharmacy, University of Eastern Finland, Yliopistonranta 1, FI-70210 Kuopio, Finland. Experimental design.; h Senior Researcher, Kuopio Research Centre of Geriatric Care, School of Pharmacy, Faculty of Health Sciences, University of Eastern Finland, Yliopistonranta 1, FI-70210 Kuopio, Finland. Proofread and critically revised the manuscript, experimental design.; i Professor, Institute of Dentistry, University of Eastern Finland, Department of Oral and Maxillofacial Surgery, Kuopio University Hospital, Kuopio, Finland. Statistical analyses, cowrote and critically revised manuscript.

**Keywords:** functional ability, oral health, oral hygiene, home care, older people

## Abstract

**Purpose:**

To describe oral health and hygiene in old home care clients and investigate how functional ability was associated with them.

**Materials and Methods:**

This study employed part of the baseline data of a multidisciplinary intervention study of 269 home care clients aged ≥75 years, living in Eastern and Central Finland. Structured interviews were used to measure ability to function in activities of daily living (ADL), instrumental activities of daily living (IADL), comorbidity (functional comorbidity index, FCI), depression (geriatric depression scale, GDS-15), cognitive function (mini-mental state examination, MMSE), nutritional status (mini nutritional assessment, MNA) and numbers of prescription drugs used. Clinical oral examination was included.

**Results:**

The majority of participants were at least moderately dependent on support for ADL. Of the examined, 46% were edentulous and average number of teeth was 8.4. Dental plaque in ≥ 20% of teeth present was detected in 74%, bleeding on probing in ≥ 25% of teeth examined in 75%, and caries in 30% of the dentate participants. In multivariate analyses, better functional ability (ADL) was statistically significantly associated with lower occurrence of dental plaque in ≥ 20% of teeth present. Better functional ability (ADL) and higher number of teeth were associated with lower occurrence of bleeding on probing in ≥ 25% of teeth examined and higher number of teeth with plaque with higher occurrence of bleeding on probing.

**Conclusion:**

Impaired functional ability is an important determinant of poor oral health and hygiene among old home care clients.

All over the world, the average lifespan of humans is getting longer and the proportion of older people in the population is growing.^2,24^ In addition, older people retain more natural teeth and are at increased risk of oral diseases and have growing needs for oral care.^29^ In previous studies oral health has consistently been described as a statistically significant determining factor for quality of life, as it affects self-esteem and the ability to communicate effectively, and has an impact on social relationships and well-being.^23^

Poor oral hygiene is an important risk factor for oral diseases in old people, owing to physical disorders as well as cognitive and social impairments.^8,29^ Most studies concerning the association of poor functional ability with oral condition have been limited either to institutionalised older patients^25^ or independent, home-dwelling older adults.^12^ They show that institutionalised and home-dwelling older people with lower functional ability have significantly poorer oral hygiene. However, information is limited concerning home-dwelling older people receiving home care.

In Finland, most home care, especially for people with poor health and functioning, is run by municipalities. In addition, older people can purchase home care services from private firms.^19^ Nowadays, although it is possible to choose from a range of home care services including mobile dental services, resources for the oral care of old people tend to be scarce and cooperation with nursing staff does not work well in practice.^29^ Due to the rapid increase in retention of natural teeth, which is expected to continue, the oral health of older adults is changing considerably. In order to improve the oral health of care-dependent older people living at home, we need timely data on their oral health status.

The aim of this study was to describe oral health and hygiene in a group of old home care clients and to study how functional ability was associated with these conditions.

## MATERIALS AND METHODS

### Study Design

This study employed part of the baseline data of a wider multidisciplinary intervention study with a population-based sample of 300 home care clients aged 75 years or over living in Eastern and Central Finland (NutOrMed-Nutrition, Oral Health and Medication) gathered in 2013.^30^ The intervention group was composed of a random sample of n = 250 home care clients from Community I (105,141 inhabitants). The control group comprised of a randomised sample of home care clients (n = 75) from Community II (20,224 inhabitants) and a total sample of n = 115 home care clients from Community III (7524 inhabitants). The intervention group was big enough to allow us to get a random sample, but the other two communities were smaller, and therefore, both communities were needed as control groups to maximise the number of controls. For the same reason, all the home care clients in Community III had the possibility to participate (total sample). To avoid contamination, the intervention group was situated approximately 100 km away from the communities of the control groups.

Randomisation inside Communities I and II was done with a coded list of home care clients and an SPSS random sample tool. The sample size calculation was estimated based on mini nutritional assessment (MNA) with the objective to detect an increase in MNA of 0.9 in the intervention group. In our previous study, the standard error of the mean change in MNA was 0.36 and the standard deviation, 3.0.^22^ The former study had a population (risk of malnutrition) comparable to the one in this study, so it was assumed that the standard error and standard deviations are also comparable. Furthermore, a power of 80% and a two-sided alpha of 0.05 were used. Based on these assumptions, 80 people were needed in each group. In all, 440 participants were selected and home care nurses provided written and oral information about the study to the clients or their care-givers. A total of 300 home care clients or their proxies initially gave written consent for the study, after which, 21 refused to participate, 4 died and 3 moved to another residence. There were no exclusion criteria.

**Fig 1:**
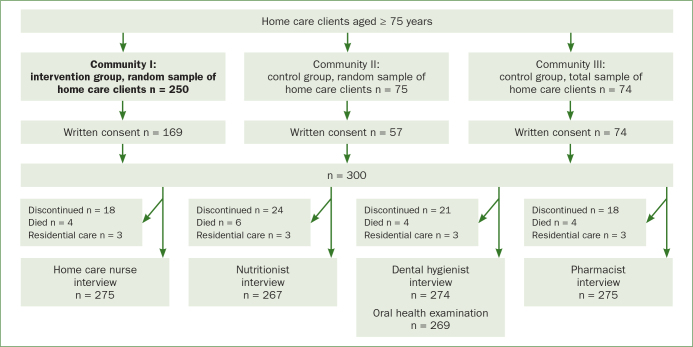
Sample of Nutrition, Oral health and Medication (NutOrMed) study at baseline in 2013.

The data used in this study were collected at baseline by interviews or clinical oral examinations carried out by a home care nurse, a nutritionist, a pharmacist and a dental hygienist at the participant’s home. Trained home care nurses ascertained the participants’ sociodemographic factors, health-related behaviour (smoking and use of alcohol), functional ability, depression and cognitive functions; a nutritionist evaluated status of nutrition and eating habits; a dental hygienist asked about oral health-related behaviour and examined oral health; and a pharmacist interviewed about their use of prescription drugs. Due to the separate interviews and clinical oral examination, the numbers of participants in different phases of the study varied (Fig 1), the eligible sample size for this study being n = 269. If the subject was cognitively impaired with poor judgement capability and was unable to reply correctly, the decision on participation in the research was made by a proxy and data collection was complemented by an interview of a caregiver or the home care nurse carrying out the interview. Of the participants, 244 participants answered the questions of the interviews by themselves, 15 needed some help from a caregiver or a nurse, and 10 participants were cognitively unable to answer the questions by themselves and all the answers were given by the caregiver or the home care nurse.

### Outcome: Oral Health and Hygiene

Before the clinical oral examination, the participants were interviewed about self-reported oral health and oral health-related behaviour by three trained dental hygienists based on written, patterned instructions. Participants were asked about the use of removable dental prostheses, toothbrushing frequency and the need for antibiotic prophylaxis before dental treatment. If there was a need for antibiotic prophylaxis, periodontal measurements were excluded from the clinical examination. Five participants who attended the interview refused to participate in the clinical oral examination which was carried out according to World Health Organization (WHO) guidelines^32^ by using a mouth mirror, a WHO periodontal probe and a headlamp with the participant either sitting or lying down.

If removable dental prostheses were present (partial or full), their cleanliness was recorded as ‘good’ or ‘poor’ denture hygiene. The number, location and condition of each tooth present was recorded (sound, satisfactory filling, fractured with no caries, carious, root caries, root without caries, missing or unable to be examined). Dichotomous variables indicating edentulousness/not and those having ≥ 20 teeth /not were created. Occurrence of decayed teeth was defined as having at least one carious tooth or root. Dental plaque was registered from each tooth according to the modified Silness and Löe index^27^ (recorded as none, found at the gingival margin only, also found elsewhere or not able to examine/tooth missing), and was described as the number of teeth with plaque. Based on this variable, we created a dichotomous measure for the occurrence of plaque, which was categorised as: 0, dental plaque on fewer than 20% of teeth present; 1, dental plaque on ≥ 20% of teeth present. This cut-off value has been considered to function well in adults and values below the cut-off indicate good oral hygiene.^1,32^ Tooth mobility was defined with one finger and an instrument and registered as none, 1–2 teeth with mobility, or 3 or more teeth with mobility. Presence of gingival bleeding on probing was also registered from each tooth except wisdom teeth and was recorded as yes, no or unable to be examined/tooth missing. A dichotomous measure was created for occurrence of bleeding on probing: 0, bleeding on probing in fewer than 25% of teeth present; 1, bleeding on probing in ≥25 % of teeth examined. According to Lang and Tonetti,^14^ individuals with mean bleeding on probing (BOP) percentages more than 25% should be considered to be at high risk for periodontal breakdown.

### Functional Ability

Functional ability was measured as functioning in the activities of daily living (ADL) and instrumental activities of daily living (IADL). ADL scores were evaluated by the Barthel index,^31^ which includes 10 factors relating to the need for assistance. Total scores from 0 to 100 represent conditions between total dependence on external assistance (0) and complete independence (100). IADL was assessed by the Lawton and Brody scale,^15^ which includes eight items (overall scores from 0 to 8, higher scores indicating better functioning).

### Confounding Factors

Cognitive status was assessed with the mini-mental state examination (MMSE) on a scale from 0 to 30, scores 18–24 indicating mild cognitive impairment and scores < 17 severe cognitive impairment^3^ and depressive symptoms with 15-item geriatric depression scale (GDS-15), which is a validated tool.^26^ In this study, GDS-15 scores were categorised into two classes: scores of 0−5 and 6−15.

The MNA^10^ was performed to evaluate nutritional status. The top score of the MNA test is 30; scores 24.0–30.0 indicate normal nutritional status, 17.0–23.5 scores a risk of malnutrition and 0–16.5 scores indicate malnutrition.

All prescription and non-prescription drugs as well as complementary and alternative medicine supplements taken regularly or as needed (within the last week) were recorded. Additionally, participants’ medication lists, drug packages and prescriptions were collected to sum up the information concerning drug use.

Data were further complemented by information concerning medical conditions, obtained from patient files. Comorbidity was computed using a modified version of the functional comorbidity index.^9^ Data of the following medical conditions were gathered at the start of the study by a physician specialised in geriatrics according to participants’ patient files and previous diagnoses: rheumatoid arthritis and other inflammatory connective tissue diseases, osteoporosis, diabetes, chronic asthma or chronic obstructive pulmonary disease, coronary artery disease, heart failure, myocardial infarctions, stroke, depressive disorder, visual impairment, Parkinson’s disease, multiple sclerosis and obesity (BMI >30). The number of conditions were added together, each one worth 1 point and higher scores representing higher comorbidity.

Information about functional ability (ADL, IADL), cognition (MMSE) and depression (GDS-15) were controlled and supplemented for all participants by information from family members or the home care nurse carrying out the interview. The home care nurses took care of each home care client on a daily basis and were trained by an experienced nutritionist (IN) from the University of Eastern Finland. The study protocol of the NutOrMed study was approved by the Research Ethics Committee of the Northern Savo Hospital District, Kuopio, Finland.

### Statistical Analysis

Baseline characteristics of the participants are presented as proportions for categorical variables and as means with standard deviations for continuous variables. Due to the skewed distributions of functional ability, comorbidity, depression, cognition and nutrition scores, the statistical significance of differences in means according to oral health were tested by Kruskal–Wallis or Wilcoxon Two-Sample tests.

Age- and gender-adjusted logistic regression analyses were first performed in order to examine associations of functional ability (ADL and IADL scores) separately with edentulousness, having ≥ 20 teeth, good denture hygiene, and occurrence of any decayed teeth, teeth with dental plaque in 20% or more of teeth present or teeth with BOP in 25% or more of teeth examined. Associations were further adjusted for cognitive function (MMSE), depression (GDS-15), comorbidity (FCI), nutritional status (MNA), number of drugs and clinically determined oral health and oral hygiene relevant to the outcome. IADL was left out of these further adjusted analyses because of high correlation (c = 0.68) with ADL.

## RESULTS

The mean age of the 269 home care clients was 84.7 (SD 5.5) years and 74% of them were female. Mean duration of education was 8.2 years (SD 3.4). Over half (54%) of the participants had ≤ 90 ADL scores, indicating a moderate dependence on support in activities of daily living. Accordingly, over two-thirds (73%) of participants had ≤ 6 IADL scores, meaning low function or high dependency. Of the participants, 66% had less than 6 points in the GDS-15 screening, a potentially depressed individual, and 41% of the participants had less than 24 points in the MMSE scores, indicating mild cognitive impairment. In MNA screening, 85% of the participants had scores of 23.5 or less, indicating a risk of malnutrition or malnutrition (Table 1). One-fifth (18%) of the participants had experienced occasional or continuous difficulties to maintain adequate oral hygiene by themselves. Only 3.2% of the respondents smoked and 8.8% used alcohol weekly.

**Table 1:** Characteristics of the participants (n = 269)

	n	%	Mean	SD
Gender				
female	198	74		
male	71	26		
Age (years)			84.7	5.5
75–84.9	150	56		
≥ 85	119	44		
Educational level (years)			8.2	3.4
Activities of daily living (ADL) score^a^	83.3	19.5		
0–20	3	1		
21–60	36	14		
61–90	100	39		
91–99	54	21		
100	63	25		
(Missing n = 13)				
Instrumental activities of daily living (IADL) score^b^			4.6	2.4
	0–6	185	73	
	7–8	69	27	
(Missing n = 15)				
Functional comorbidity (FCI) score^c^			2.9	1.9
15-item geriatric depression scale (GDS-15) score^d^			4.8	3.1
0–5	169	66		
≥ 6	86	34		
(Missing n = 14)				
Mini-mental state examination (MMSE) score^e^			23.1	5.3
0–23	104	41		
24–30	147	59		
(Missing n = 18)				
Mini nutritional assessment (MNA) score^f^			21.7	2.7
0–16.5	12	5		
17–23.5	211	80		
24–30	38	15		
(Missing n = 8)				
Number of drugs used^g^			10.2	3.9
0–5	30	11		
6–9	91	34		
≥10	147	55		
(Missing n = 1)				
Smoking				
has never smoked	174	69.6		
quitted	68	27.2		
occasional smoker	1	0.4		
daily smoker	7	2.8		
(Missing n = 19)				
Frequency of alcohol use				
never	165	66.0		
once a month or less often	47	18.8		
2 to 4 times per month	16	6.4		
2 to 3 times per week	12	4.8		
at least 4 times per week	10	4.0		
(Missing n = 19)				

SD, standard deviation; ^a^ Barthel index, range 0–100, higher scores indicate better functional ability; ^b^ range 0–8, higher scores indicate better functional ability; ^c^ range 0–18, higher sum score represent greater comorbidity; ^d^ range 0–15, ≥6 scores indicate a potentially depressed individual; ^e^ range 0–30, higher scores indicate better function; ^f^ range 0–30, higher scores indicate better nutrition; ^g^ the number of regularly or as needed (within the last week); prescription or over-the-counter drugs.

Of those examined, 46% were edentulous and the average number of teeth present among the dentate subjects was 15.6 (SD 7.6). Overall, 69% of the participants wore dentures; among them, poor denture hygiene was detected in 41% and a need for denture repair in 58%. Of the dentate participants, 30% had at least one decayed tooth and root caries was observed among 8% of the dentate participants. Teeth with plaque in 20% or more of teeth present were detected in 74% of those dentate examined. BOP in 25% or more of teeth examined was detected in 75% of the dentate participants (Table 2).

**Table 2:** Clinically determined oral health of the participants

	mean (SD)	n (%)
ALL (n = 269)		
Denture status		
Edentulous, no dentures		8 (3)
Edentulous, full dentures		115 (43)
Dentate, full/partial dentures		71 (26)
Dentate, no dentures		75 (28)
Number of teeth present	8.4 (9.6)	
Number of teeth ≥ 20		51 (19)
DENTURE WEARERS (n = 186)		
Need for repair		107 (58)
Poor hygiene		76 (41)
DENTATE (n = 146)		
Number of teeth	15.6 (7.6)	
Number of teeth ≥ 20		51 (35)
Condition of teeth		
Decayed teeth	0.8 (1.8)	44 (30) ^a^
Teeth with root caries	0.1 (0.6)	12 (8) ^a^
Teeth with plaque	9.5 (8.4)	112 (77) ^a^
Teeth with plaque ≥ 20%		108 (74) ^b^
Mobile teeth	0.1 (0.4)	18 (12) ^a^
Condition of periodontium ^c^		
Teeth with BOP ^d^	9.6 (8.4)	86 (84) ^a^
Teeth with BOP ≥ 25%		77 (75) ^b^

^a^ Any = at least one tooth with caries, root caries, dental plaque, mobility or bleeding on probing; ^b^ Of all teeth; ^c^ Among dentate persons with no need for antibiotic prophylaxis during dental treatment (n = 103); ^d^ BOP, bleeding on probing.

In general, participants with poorer oral health had poorer functional ability, cognition, health or nutritional status. Those who had teeth with plaque in ≥ 20% of teeth present, decayed teeth or teeth with BOP in ≥ 25% of teeth examined had statistically significantly poorer functional ability (either measured by ADL or IADL) than their counterparts. In addition, those who did not have ≥ 20 teeth had greater comorbidity (FCI) and those who had teeth with dental plaque had poorer cognition (MMSE) (Table 3).

**Table 3:** Mean (SD) scores of functional ability (ADL^a^, IADL^b^), cognition (MMSE), comorbidity (FCI) and nutritional status (MNA) according to oral health

	Activities of daily living (ADL) score^a^	Instrumental activities of daily living (IADL) score^b^	Functional comorbidity (FCI) score^c^	15-item geriatric depression scale (GDS-15) score^f^	Mini-mental state examination (MMSE) score^d^	Mini nutritional assessment (MNA) score^e^
	Mean (SD)					
Denture status						
Edentulous	83.4 (20.1)	4.4 (2.3)	3.1 (1.8)	5.1 (3.2)	23.0 (5.3)	21.6 (2.8)
Dentate, full/partial dentures	84.0 (17.6)	5.0 (2.4)	2.8 (1.9)	4.7 (3.0)	23.4 (4.7)	21.9 (2.5)
Dentate, no dentures	82.5 (19.0)	4.5 (2.5)	2.6 (1.8)	4.3 (2.8)	23.2 (5.9)	21.7 (2.9)
	p = 0.767*	p = 0.298*	p = 0.125*	p = 0.270*	p = 0.665*	p = 0.766*
Number of teeth						
<20	82.9 (20.1)	4.5 (2.4)	3.0 (1.9)	4.9 (3.0)	23.0 (5.2)	21.7 (2.8)
≥ 20	85.0 (16.7)	4.8 (2.3)	2.4 (1.8)	4.4 (3.2)	23.6 (6.0)	21.7 (2.6)
	p = 0.865	p = 0.424	p = 0.043	p = 0.210	p = 0.243	p = 0.389
Oral hygiene						
Teeth with plaque						
≥ 20% of all teeth	80.4 (18.9)	4.4 (2.5)	2.8 (1.9)	4.6 (2.9)	22.6 (5.6)	21.6 (2.8)
<20% of all teeth	91.1 (14.1)	5.6 (2.1)	2.3 (1.9)	4.3 (3.1)	25.3 (3.9)	22.3 (2.5)
	p <0.001	p = 0.007	p = 0.091	p = 0.505	p = 0.015	p = 0.291
Condition of teeth						
Decayed teeth						
≥ 1	80.0 (17.4)	4.5 (2.4)	2.8 (2.0)	4.8 (3.0)	22.4 (6.2)	21.7 (2.9)
None	84.8 (18.6)	4.8 (2.4)	2.7 (1.8)	4.4 (2.9)	23.7 (4.9)	21.9 (2.7)
	p = 0.045	p = 0.336	p = 0.933	p = 0.480	p = 0.373	p = 0.834
Teeth with root caries						
≥ 1	77.3 (16.1)	4.5 (2.3)	1.6 (1.0)	4.8 (2.5)	23.3 (5.4)	20.8 (3.1)
None	83.8 (18.5)	4.7 (2.4)	2.8 (1.9)	4.5 (3.0)	23.3 (5.4)	21.9 (2.7)
	p = 0.081	p = 0.688	p = 0.052	p = 0.562	p = 0.985	p = 0.163
Mobile teeth						
≥ 1	76.0 (20.1)	4.2 (2.5)	2.5 (1.7)	5.2 (2.7)	24.7 (3.4)	21.0 (3.4)
None	84.2 (17.9)	4.8 (2.4)	2.7 (1.9)	4.4 (2.9)	23.1 (5.6)	21.9 (2.6)
	p = 0.930	p = 0.395	p = 0.731	p = 0.199	p = 0.351	p = 0.521
Condition of periodontium						
Teeth with BOP						
≥ 25% of all teeth	77.9 (20.1)	4.3 (2.4)	2.2 (1.5)	4.2 (2.7)	22.8 (5.6)	21.5 (2.9)
< 25% of all teeth	91.2 (13.0)	5.4 (2.2)	2.0 (1.7)	4.2 (3.0)	24.8 (5.2)	22.6 (3.4)
	p = 0.003	p = 0.060	p = 0.411	p = 0.887	p = 0.078	p = 0.119

SD, standard deviation; BOP, bleeding on probing; a Barthel index, range 0–100, higher scores indicate better functional ability; b range 0–8, higher scores indicate better functional ability; c higher sum score represents greater comorbidity; d, range 0–15, higher scores indicate potentially depressed individual; e range 0–30, higher scores indicate better function; f range 0–30, higher scores indicate better nutrition; * based on Kruskal–Wallis tests, otherwise Wilcoxon Two-Sample tests.

In age- and gender-adjusted logistic regressions, better functional ability (higher scores in ADL; Barthel index and IADL index) associated significantly with occurrence of plaque in ≥ 20% of teeth present and BOP in ≥ 25% of teeth examined (Table 4) but not with the other outcomes studied. In the further adjusted analyses, subjects with better functional ability (higher scores in ADL; Barthel index) had fewer teeth with plaque in ≥ 20% of teeth present (odds ratio (OR) = 0.95, 95%CI = 0.9–1.0, Table 4). In addition, those with a higher number of teeth with plaque had greater odds of having BOP in ≥ 25% of teeth examined (OR = 1.18, 95%CI 1.1–1.3) and those with better functional ability (higher scores in ADL; Barthel index) and a higher number of teeth had lower odds (OR 0.94, 95%CI 0.9–1.0 and OR 0.87, 95%CI 0.8–1.0; Table 5). Sensitivity analyses including toothbrushing frequency in the models was conducted, but we found neither any change in the results nor an association between toothbrushing frequency and number of teeth with dental plaque or BOP (data not shown). In these adjusted analyses, functional ability (measured by ADL; Barthel index) was not associated with being edentulous or having ≥ 20 teeth, poor denture hygiene or dental caries (data not shown).

**Table 4:** Age- and gender-adjusted associations ORs (odds ratio) with 95% CI (= 95% confidence interval) of oral health and hygiene by functional ability: activities of daily living (ADL^a^) and instrumental activities of daily living (IADL^b^)

Oral health and hygiene	ADL^a^			IADL^b^		
	OR	95% CI	p value	OR	95% CI	p value
Edentulous	1.00	0.99–1.01	0.824	0.96	0.86–1.07	0.498
	(n = 256)			(n = 254)		
Number of teeth ≥ 20	1.01	0.99–1.02	0.497	1.05	0.91–1.20	0.508
	(n = 256)			(n = 254)		
Good denture hygiene	1.10	0.99–1.02	0.521	1.10	0.96–1.27	0.181
	(n = 176)			(n = 173)		
Occurrence of decayed teeth	0.98	0.96–1.00	0.093	0.92	0.79–1.07	0.270
	(n = 140)			(n = 139)		
Plaque in ≥ 20 % of teeth present	0.95	0.92–0.99	0.014	0.80	0.67–0.95	0.010
	(n = 140)			(n = 139)		
Teeth with BOP in ≥ 25 % of teeth present	0.95	0.91–0.98	0.005	0.82	0.67–1.01	0.064
	(n = 99)			(n = 97)		

^a^ Barthel index, range 0–100, higher scores indicate better functional ability; ^b^ range 0–8, higher scores indicate better functional ability.

**Table 5:** Association between patient characteristics and the presence of dental plaque in 20 % or more of teeth present from multivariate logistic regression

	Teeth with plaque in 20 % or more of teeth present (n = 130)		
	OR	95% CI	p value
Gender (ref. male)	0.69	0.22–2.11	0.512
Age (years)	0.97	0.89–1.05	0.426
ADL^a^	0.95	0.91–1.00	0.031
FCI ^b^	1.18	0.89.1.58	0.251
GDS-15 ^c^	0.97	0.83–1.14	0.689
MMSE ^d^	0.93	0.84–1.03	0.138
MNA ^e^	0.97	0.80–1.16	0.814
Number of drugs ^f^	0.92	0.79–1.08	0.317
No removable dentures (ref. yes)	1.83	0.53–6.33	0.340
Number of teeth	0.95	0.88–1.03	0.186
Number of decayed teeth	1.52	0.83–2.80	0.173

OR, odds ratio; 95%CI, 95% confidence interval. ^a^ Barthel index, higher scores indicate better functional ability; ^b^ functional comorbidity index, higher sum score represent greater comorbidity; ^c^ GDS-15, 15-item geriatric depression scale, higher scores indicate a potentially depressed individual; ^d^ MMSE, mini-mental state examination, higher scores indicate better cognition; ^e^ mini nutritional assessment test, higher scores indicate better nutrition; ^f^ The number of regularly or as needed (within last week) prescription or over-the-counter drugs.

## DISCUSSION

In this study, we analysed oral health and hygiene among very old home care clients, and how functional ability was associated with these factors. In general, ADLs, cognition and nutritional status proved to be poor. In addition, about half of the participants were edentulous and two-thirds of them had removable dentures, which were often dirty or in poor condition. Oral hygiene was generally poor and periodontal problems were common. However, dental caries was found only in one-third of participants and root caries occurred rarely. In general, there was a tendency that those with poorer functional ability also had poorer oral health. In particular, poor functional ability measured by ADL was associated with a higher number of teeth with plaque and a higher occurrence of BOP.

Although the sample examined was of relatively small size, the population-based design and the multidisciplinary approach are strengths of the present study. Random samples from the three municipalities indicated that the study population comprehensively represented the older range of people receiving homecare. Finland is ethnically homogenous, and home care provided by municipalities is organised similarly in all regions. In addition, the present study had no exclusion criteria regarding age, morbidity or the cognitive status of the home care clients. The answers obtained from persons with cognitive decline were checked with their proxy, family member or nurse. The data collection was performed by a multiprofessional team that included researchers and clinicians from several fields such as geriatrics, dentistry, nutrition and pharmacy.

Another strength of this study is that we used two commonly used and validated instruments to measure functional ability. ADLs are activities in which people engage on a day-to-day basis. These are everyday personal care activities that are fundamental to caring for oneself and maintaining independence. IADLs are activities related to independent living and are valuable for evaluating persons with early-stage disease, both to assess the level of disease and to determine the person’s ability to care for himself or herself. In further adjusted analyses, ADL was chosen instead of IADL because we found it more relevant for maintaining oral health and hygiene. We also used several validated instruments to measure cognition, morbidity, nutrition and oral health. There is a considerable amount of previous research on health and functional ability of institutionalised older patients or older people living independently at home,^5,13,23,18^ but few studies have focused on home-dwelling older people receiving home care.^7^ In particular, clinical examinations of these populations are rare.

One limitation of our study is its cross-sectional design, which does not allow any causal inferences to be drawn. Another limitation is that some of the data were collected by several nurses and dental hygienists, which could affect internal reliability. However, all the nurses and dental hygienists were trained by the same person, thus reducing inter-examiner variability. In addition, the clinical examination was undertaken by a dental hygienist in the participant’s home. Examinations performed at a dental clinic under optimal conditions (ie, with better lightning and the possibility of taking radiographs, could have improved the validity and reliability of the data).^6^ Lack of statistical power may have been the reason behind the non-statistically significant associations found in this study due to relatively small data. This was particularly relevant regarding the more complicated analyses of periodontal condition, for which measures were available only for a reduced number of participants.

In our study, the participants with more needs for supportive care (ie, poorer functional ability) had statistically significantly more teeth with plaque. Dental plaque was detected in most participants and poor denture hygiene in almost half of the participants, suggesting that oral hygiene was not maintained effectively. Previous research^16^ has shown that home-dwelling older people with lower functional ability were less likely to brush their teeth twice a day, less likely to use toothpaste and less likely to have good oral hygiene. Research also shows that older people with cognitive impairments, poor oral hygiene, reduced functional abilities and a substantial need for daily support show more active caries lesions. This has been shown to be the main threat to the oral health of older people.^11,29^ Poorer functional ability was also associated with the prevalence of BOP in this study. This is in line with the results of Strömberg et al (2012),^29^ who found that participants (aged 65 to 101 years) with substantial needs for supportive care had higher levels of BOP than those with moderate supportive needs.

Our result that poorer functional ability associated with presence of plaque and BOP is plausible, since those are the primary changes one can imagine as a result of poor self-care. Other outcomes included in this study need longer time to progress and no clear associations with poor functional ability was seen. Our study found no association of being edentulous or having ≥ 20 teeth with functional ability. The same goes for comorbidity, nutritional status, cognitive function or number and prescription drugs, although other studies have shown that poorer status in disability, physical health and nutrition are associated with edentulousness.^28^ Similarly to our findings, Strömberg et al (2012)^29^ found that individuals with moderate and substantial needs for supportive care had no differences in numbers of teeth. There was also no statistically significant difference between these two groups as regards being edentulous or having removable dentures.

Even though poor functional ability seems to contribute to poor oral health of the participants, it is probably not the only reason. Considering the frame of this generation in Finland, the mean age of the participants (84.7 years) may explain the fact that 43% of the participants had a full denture and 28% had a partial denture. The fact that dental caries was only found in one-third of the participants may be due to the high prevalence of edentulousness and also to the low number of teeth among the dentate; 81% of the participants had fewer than 20 remaining teeth. Another reason could be that dental cavities could be considered as long-term effects of reduced functional ability, and in our study it was not known when functional ability started to deteriorate. However, having decayed teeth associated statistically significantly with poorer functional ability measured by ADL in the unadjusted analyses. This supports the finding that those with poorer functional ability generally tended to have poorer oral health.

Of the participants, one-fifth experienced occasional or continuous difficulties in performing adequate oral hygiene by themselves. Niesten et al^20^ found that for older people living in regular day care centres and assisted-living homes, adhering to daily oral hygiene routines was important, and especially for those who felt themselves to be weak, because it helped them to maintain their sense of autonomy and feelings of self-worth. However, participants were lacking the support they needed, or it was hard to get. Nurses were too rushed and did not always clean or rinse dentures properly, so that they remained dirty or tasted of soap. Nursing teams along with health professionals play a key role in promoting oral health by detecting dental diseases early, supporting oral hygiene and adequate nutrition, and preventing discomfort. Although the integration of oral healthcare into day-to-day care seems to be a major problem due to a multitude of barriers, such as lack of adequate dental knowledge, lack of support from a dentist or negative emotions towards oral health,^4^ compassionate care and instructed training programmes for nurses aimed at improving oral care support are proven to increase their willingness to support residents with their oral care.^17^ In addition, dental hygienists could more actively take part in nursing teams and thus enhance collaboration between home care nurses and oral healthcare.

Old people at risk of becoming socially and economically marginalised pose a major challenge for promotion of oral health, as in the future more dependent individuals with more substantial disabilities will be living at home. Maintaining good oral health in vulnerable older people needs to be given high priority in the future, as oral diseases may lead to pain, infection and tooth loss and may compromise chewing capacity, leading to impaired nutritional status.^16,21^

## CONCLUSION

Impaired functional ability was associated with poor oral hygiene and BOP, which may predispose old people to more severe health-related problems (Table 6).

**Table 6:** Association between patient characteristics and presence of teeth with bleeding on probing (BOP) in 25% or more of teeth present from multivariate logistic regression

	Teeth with BOP in 25 % or more of teeth present (n = 90)		
	OR	95% CI	p value
Gender (ref. male)	0.46	0.10–2.12	0.316
Age (years)	0.95	0.85–1.06	0.378
ADL^a^	0.94	0.90–0.98	0.004
FCI ^b^	0.75	0.43–1.29	0.298
GDS-15 ^c^	0.82	0.62–1.09	0.167
MMSE ^d^	1.04	0.90–1.22	0.572
MNA ^e^	0.89	0.66–1.19	0.435
Number of drugs ^f^	0.95	0.77–1.18	0.656
Removable dentures (ref. no)	2.12	0.27–16.45	0.472
Number of teeth present	0.87	0.76–1.00	0.046
Number of teeth with plaque	1.18	1.07–1.30	0.001

OR, odds ratio, 95%CI, 95% confidence interval. ^a^ Barthel index, higher scores indicate better functional ability; ^b^ functional comorbidity index, higher sum score represent greater comorbidity; ^c^ GDS-15, 15-item geriatric depression scale, higher scores indicate a potentially depressed individual; ^d^ MMSE, mini-mental state examination, higher scores indicate better cognition; ^e^ mini nutritional assessment test, higher scores indicate better nutrition; ^f^ The number of regularly or as needed (within last week) prescription or over-the-counter drugs.
